# Minocycline does not affect long-term potentiation in the anterior cingulate cortex of normal adult mice

**DOI:** 10.1186/s12990-015-0025-2

**Published:** 2015-05-02

**Authors:** Qian Song, Ming-Gang Liu, Min Zhuo

**Affiliations:** Center for Neuron and Disease, Frontier Institutes of Science and Technology, Xi’an Jiaotong University, Xi’an, 710049 China; Department of Physiology, Faculty of Medicine, University of Toronto, 1 King’s College Circle, Toronto, Ontario M5S 1A8 Canada; Department of Anatomy, Histology and Embryology, Institute of Medical Sciences, Shanghai Jiao Tong University School of Medicine, Shanghai, 200025 China

**Keywords:** Minocycline, Multi-electrode array, Presynaptic long-term potentiation, Postsynaptic long-term potentiation, Anterior cingulate cortex

## Abstract

It has been reported that activated microglia plays important roles in chronic pain-related sensory signaling at the spinal cord dorsal horn. Less is known about the possible contribution of microglia to cortical plasticity that has been found to be important for chronic pain. In the present study, we used a 64-channel multi-electrode array recording system to investigate the role of microglia in cortical plasticity of the anterior cingulate cortex (ACC) in normal adult mice. We found that bath application of minocycline, an inhibitor of microglial activation, had no effect on postsynaptic LTP (post-LTP) induced by theta burst stimulation in the ACC. Furthermore, presynaptic LTP (pre-LTP) induced by the combination of low-frequency stimulation with a GluK1-containing kainate receptor agonist was also not affected. The spatial distribution of post-LTP or pre-LTP among the cingulate network is also unaltered by minocycline. Our results suggest that minocycline does not affect cingulate plasticity and neurons are the major player in pain-related cortical plasticity.

**Electronic supplementary material:**

The online version of this article (doi:10.1186/s12990-015-0025-2) contains supplementary material, which is available to authorized users.

## Introduction

Microglia is one of the major resident immune cells in the brain [1]. It is well documented that activated microglia critically contributes to a variety of physiological and pathological conditions, such as learning and memory, pain and addiction [2-5] (Additional file 1: Table S1). In the spinal cord, microglia can be rapidly activated after peripheral inflammation or nerve injury [6,7], and is actively involved in the development of pain hypersensitivity by releasing various cytokines and chemokines [8-11]. Unlike spinal cord and other brain regions, fewer studies have been reported on the possible roles of microglia in chronic pain-related cortical areas. Microglia activation in the anterior cingulate cortex (ACC) or prefrontal cortex (PFC) is shown to correlate with pain-evoked comorbid symptoms, including depression and negative aversion, as well as sensory abnormalities [12-14]. However, our previous work failed to detect any significant activation of microglial cells in the ACC or PFC in a common peroneal nerve (CPN) ligation model of neuropathic pain [15].

Long-term potentiation (LTP) is a major form of synaptic plasticity in the brain [16,17]. In the ACC, a major region for the pain perception and unpleasantness, LTP may serve as a cellular mechanism for chronic pain [18,19]. Recent studies have shown the contribution of microglia to the induction of LTP at the spinal cord dorsal horn [20-22]. Previous reports of microglia in the hippocampus demonstrate that microglia is involved in age- or β-amyloid-induced LTP impairment [23-25]. In contrast, we found that strong tetanic stimulation of Schaffer-collateral pathways that induced CA1 LTP did not affect microglial motilities in the hippocampus [26]. Therefore, it is likely that microglia plays a minor role in neuronal activity and plasticity under normal condition. As compared to the spinal cord and hippocampus, however, little information is available on microglia and LTP in the cerebral cortex (Additional file 2: Table S2).

To address this question, we used a 64-channel multi-electrode dish (MED64) recording system to test the effects of minocycline, a potent microglial inhibitor [11,12], on the induction of two forms of LTP in the adult mouse ACC: presynaptic LTP (pre-LTP) [27] and postsynaptic LTP (post-LTP) [28]. Our recent work demonstrates the co-existence of these two forms of LTP in the ACC synapses, which have different mechanisms of induction and behavioral implications [29]. In the present study, we found that neither post-LTP nor pre-LTP in the ACC was affected by minocycline treatment, indicating a minor role of microglia in cingulate plasticity.

## Methods

### Animals

The experiments were carried out on male C57BL/6 mice (6-10 weeks old, Charles River, Quebec, Canada). All animals were fed in groups of three per cage under standard laboratory conditions (12 h light/12 h dark, temperature 22-26°C, air humidity 55-60%) with *ad libitum* food and water. The experimental procedures were approved by the Institutional Animal Care and Use Committee of The University of Toronto and Xi’an Jiaotong University.

### Drugs

The chemicals and drugs used in this study were as follows: D-(-)-2-amino-5-phosphonopentanoic acid (AP5) was purchased from Sigma (St. Louis). (RS)-2-Amino-3-(3-hydroxy-5-tert-butylisoxazol-4-yl) propanoic acid (ATPA) and minocycline hydrochloride were purchased from Tocris Cookson (Bristol, UK). Drugs were prepared as stock solutions for frozen aliquots at -20°C. All these drugs were diluted from the stock solution to the final desired concentration in the artificial cerebrospinal fluid (ACSF) before immediate use.

### Brain slice preparation

The general procedures for making the ACC slices are similar to those described previously [30-32]. Mice were anesthetized with gaseous isoflurane and decapitated. The whole brain was rapidly removed and transferred to ice cold oxygenated (95% O_2_ and 5% CO_2_) ACSF containing (in mM): NaCl 124, KCl 2.5, NaH_2_PO_4_ 1.0, MgSO_4_ 1, CaCl_2_ 2, NaHCO_3_ 25 and glucose 10, pH 7.35-7.45. After cooling for about 2 min, appropriate parts of the brain were then trimmed and the remaining brain block was glued onto the ice-cold stage of a vibrating tissue slicer (Leica VT1200S). Then three coronal ACC brain slices (300 μm), after the corpus callosum meets, were cut and transferred to an incubation chamber with oxygenated ACSF at room temperature for at least 2 h.

### Preparation of the multi-electrode array probe

The procedures for preparation of the MED64 probe were similar to those described previously [31,33,34]. The MED64 probe (MED-P515A, Panasonic Alpha-Med Sciences, Japan) has an array of 64 planar microelectrodes, arranged in an 8 × 8 array, with an interpolar distance of 150 μm. Before use, the surface of the MED64 probe was treated with 0.1% polyethyleneimine (Sigma, St Louis, MO; P-3143) in 25 mmol/l borate buffer (pH 8.4) overnight at room temperature. Then, the probe surface was rinsed three times with sterile distilled water before each experiment.

### Multi-channel field potential recordings

**Figure 1:**
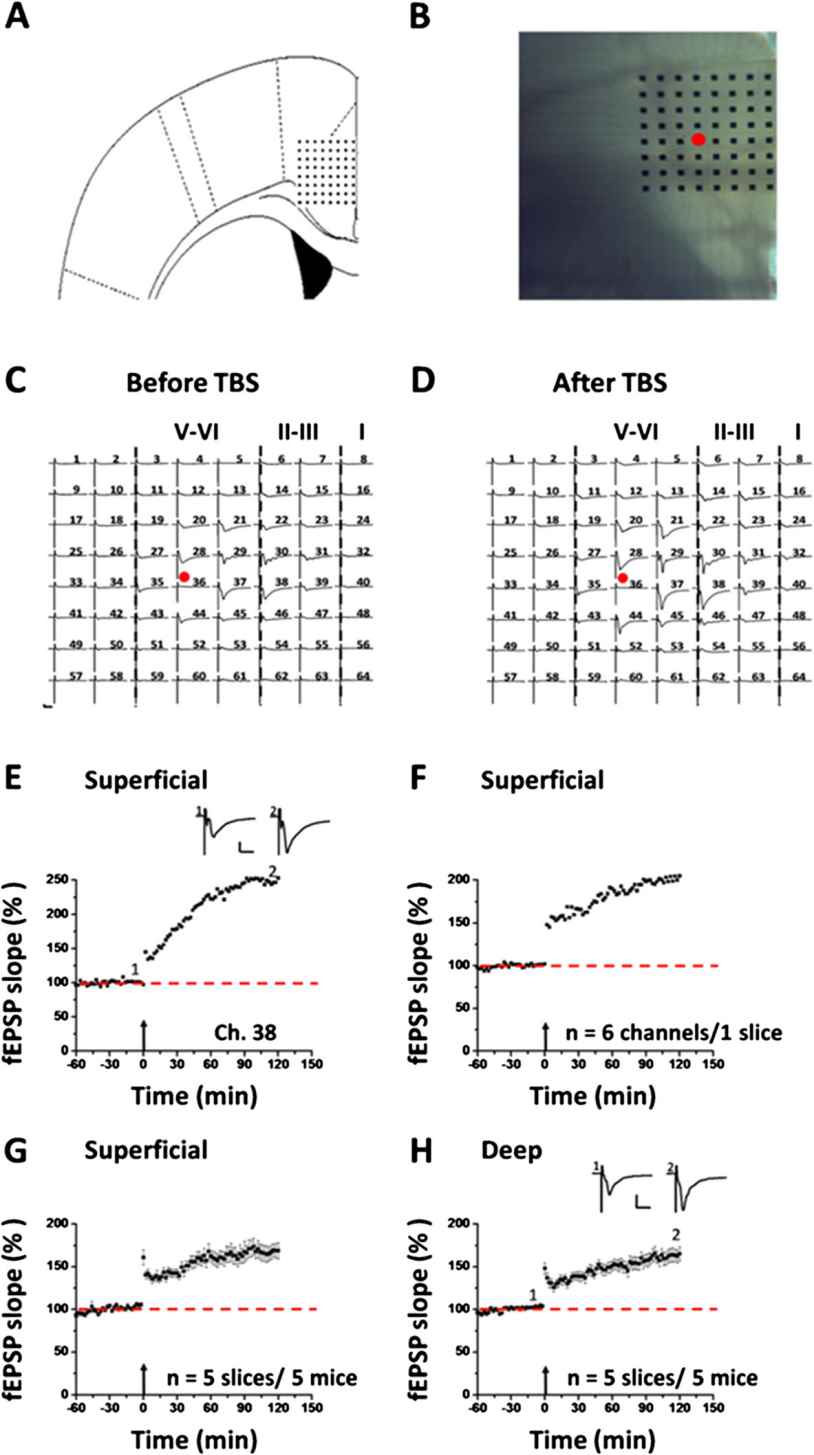
Multi-channel recordings of post-LTP in the adult mouse ACC. **A**: schematic diagram showing the location of the MED-64 probe on an ACC slice. **B**: light microscopy photograph showing the relative location of ACC slice and probe. The red circle indicates the stimulation site. **C** and **D**: an overview of multi-site synaptic responses recorded at baseline (C) and 2 h after TBS (D) in one slice. After stabilizing the baseline responses for 1 h, a TBS protocol was delivered to the deep layer and LTP was then monitored for 2 h. The red filled circle denotes the stimulated channel (Ch. 36). Vertical lines indicate the layers of the ACC slice. **E**: results of one superficial channel (Ch. 38) showing the induction of post-LTP that lasts for 2 h in one slice. **F**: summary of averaged data from 6 channels in the superficial layer of the same slice. **G**: pooled data of the superficial layer from 5 slices of 5 mice. **H**: pooled data of the deep layer from 5 slices of 5 mice. Insets in E and H are sample traces at the time points indicated by numbers in the graph. Calibration: 100 μV, 10 ms. Arrows in E-H indicate starting point of TBS application. Error bars in G and H represent SEM.

After incubation, one slice was positioned on the MED64 probe in such a way that the ACC area was entirely covered by the recording dish (Figure 1A and B) mounted on the stage of an inverted microscope (CKX41, Olympus). Once the slice was settled, a fine mesh anchor (Warner Instruments, Harvard) was carefully positioned to ensure slice stability during recording. The slice was continuously perfused with oxygenated, fresh ACSF at the rate of 2–3 ml/min with the aid of a peristaltic pump (Minipuls 3, Gilson) throughout the entire experimental period.

After a 10-15 min recovery period, one of the channels located in the deep layer (V-VI) of the ACC, from which the best synaptic responses can be induced in the surrounding channels, was chosen as the stimulation site. Monopolar and biphasic constant current pulses (10-20 μA, 0.2 ms) were applied to the stimulation site via the Mobius software and field excitatory postsynaptic potential (fEPSP) evoked at both superficial layer (II-III) and deep layer of the ACC were amplified by a 64-channel amplifier, displayed on the monitor screen, and stored on the hard disk of a microcomputer for off-line analysis. Channels in which field potentials can be induced were considered as active and their responses were sampled every 1 min. After the baseline responses were stabilized for at least 1 h, a theta burst stimulation (TBS) protocol (5 bursts at 5 Hz, 4 pulses at 100 Hz for each burst) was given 5 times (10 s interval) to induce post-LTP at the stimulation intensity which was adjusted to elicit 40-60% of the maximal response [30,31,35]. To induce pre-LTP, a low-frequency stimulation (LFS, 2 Hz for 2 min) combined with a GluK1 receptor agonist (ATPA, 1 μM, 18 min) was applied in the presence of the NMDA receptor antagonist (AP-5, 50 μM, 38 min) as described previously [27]. After LFS + ATPA or TBS, the synaptic responses were monitored for 1 h or 2 h to see the time course of pre-LTP or post-LTP. Notably, for post-LTP experiment, we always used single pulse recording, while for pre-LTP experiment, the control test pulses are paired-pulses with an interval being 50 ms.

### Data analysis

MED64 Mobius software was used for all multichannel electrophysiological data acquisition and analysis. To quantify the data, the initial slope of fEPSP was measured, normalized and presented separately in both superficial and deep layers as a percentage change from the averaged baseline level. The degree of synaptic potentiation in each experiment was shown as the value obtained at 1 h or 2 h for pre-LTP or post-LTP, respectively. For the pre-LTP data, we also calculated the paired-pulse ratio (PPR) by the following formula: PPR = the slope of the second fEPSP/the slope of the first fEPSP. We defined LTP in a channel if the response was increased by at least 20% of baseline during the entire recording period. The number of activated channels (over 20% of baseline, i.e. the amplitude goes over -20 μV) vs. LTP-showing channels was counted and expressed as the induction ratio of LTP (number of LTP-occurring channels/number of all activated channels × 100%) [30]. Channels with baseline response variation > 5% were discarded. All data are presented as mean ± SEM. The student’s t-test was used for statistical comparisons. *P* < 0.05 was considered to be statistically significant.

## Results

### Multi-channel recordings of post-LTP in the ACC circuit

Before investigating the effect of minocycline on cingulate LTP, we first mapped the network properties of post-LTP induction and distribution in the ACC of adult mice. Figure 1A and B show the location of the MED64 probe in the ACC slice. One representative example of the LTP recording is illustrated in Figure 1C and D. One channel (Ch. 36, Figure 1C and D, red circle) that located on the deep layer V-VI of the ACC was chosen as the stimulation site and fEPSPs recorded from the other channels were monitored. We found that fEPSPs could be reliably recorded from both superficial layer (II-III) and deep layer of the ACC slice (Figure 1C). After the stable baseline recording for at least 1 h, a TBS protocol (see the Method for details) was delivered to the same stimulation site to induce post-LTP. Similar to previous reports, we found that TBS resulted in stable LTP that lasted for 2 h in most of the active channels (Figure 1D). Figure 1E shows the data of one channel (Ch. 38) located in the superficial layer, with the fEPSP slope reaching 249.0% of baseline at 2 h after TBS. The averaged data from 6 channels in superficial layer of the sample slice is plotted in Figure 1F (199.9% of baseline at 2 h after TBS). In total, from 5 slices of 5 mice, we found that the mean percentage of fEPSP slope was potentiated to 168.1 ± 0.7% of baseline in the superficial layer (*P* < 0.001, paired t-test, Figure 1G). In the deep layer, we obtained similar results, with the averaged fEPSP slope being potentiated to 161.5 ± 0.7% of baseline at the end of the recording period (n = 5 slices/5 mice, *P* < 0.001, paired t-test, Figure 1H).

**Figure 2:**
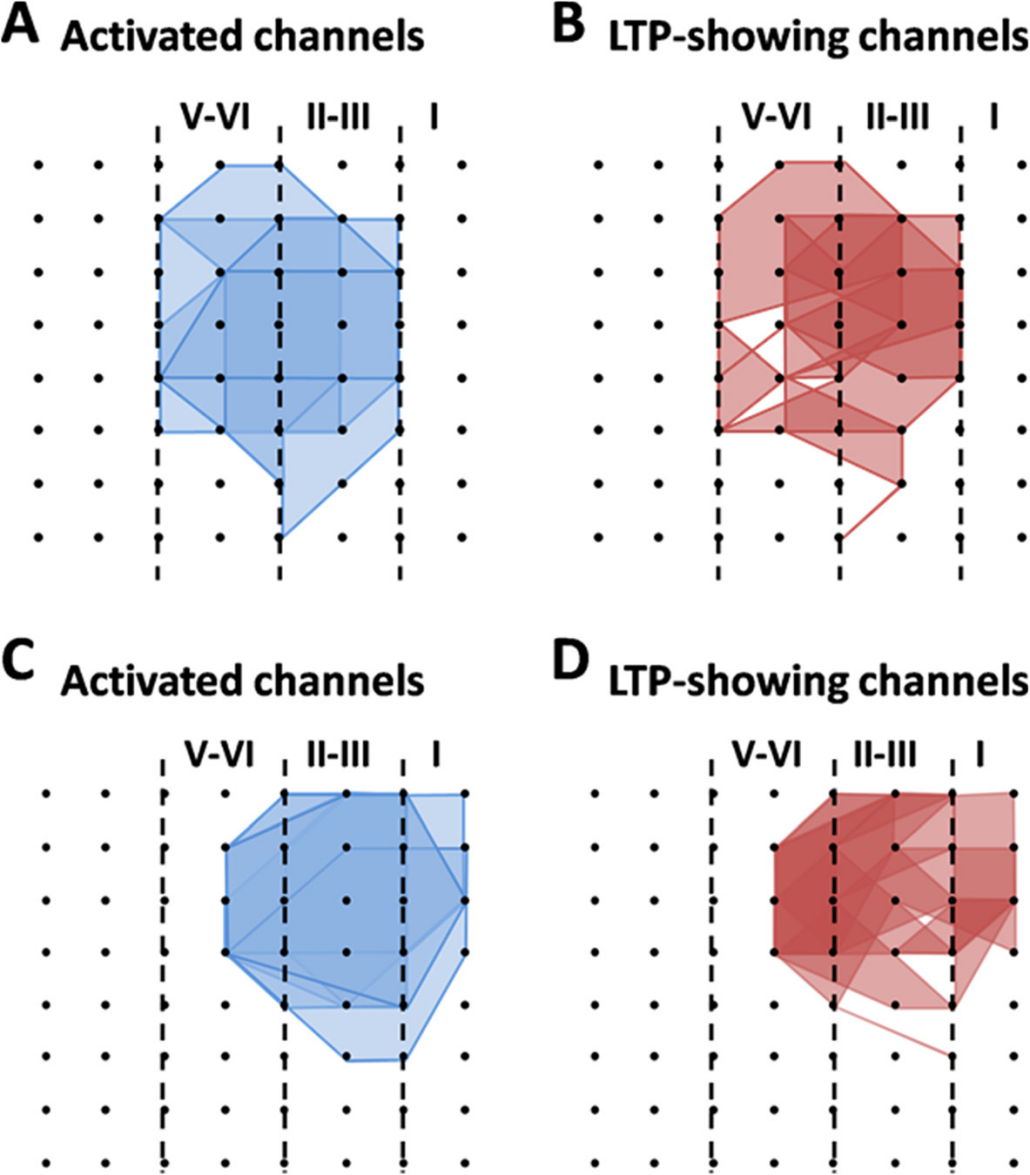
Spatial analysis of post-LTP distribution in the ACC. **A** and **B**: polygonal diagram of the channels that are activated in the baseline (blue, A) and that undergo post-LTP (red, B) in 5 slices from 5 mice. Vertical lines denote the layers in the ACC slice. Overlapped blue regions denote frequently activated channels, while overlapped red regions indicate the channels that are most likely to undergo post-LTP. Most, although not all, of the activated channels exhibited post-LTP after TBS. **C** and **D**: spatial analysis of the effects of minocycline on post-LTP distribution maps in the ACC. Shown are the polygonal graphs of activated (blue, C) and post-LTP-occurring (red, D) channels among the cingulate network when TBS is applied in the presence of minocycline (n = 7 slices/7c mice). Minocycline does not affect the number of post-LTP-showing channels in the ACC.

We next mapped the spatial distribution of the active responses and LTP-showing channels around the ACC network through a previous-established method [30]. The activated channels are displayed by the blue lines (Figure 2A) and the post-LTP-showing channels are shown by the red lines (Figure 2B). Consistent with our previous studies [31,36], TBS did not elicit LTP in every channel in the ACC. In total, 105 channels (mean ± SEM: 21.0 ± 0.4) exhibited active synaptic responses from 5 slices, with 85 channels (mean ± SEM: 17.0 ± 0.9) undergoing post-LTP. The induction ratio of post-LTP is 80.8 ± 3.3%. Also, the LTP-occurring channels were found in both layer II-III and layer V-VI (Figure 2B).

### Minocycline has no effect on post-LTP induction in the ACC

**Figure 3:**
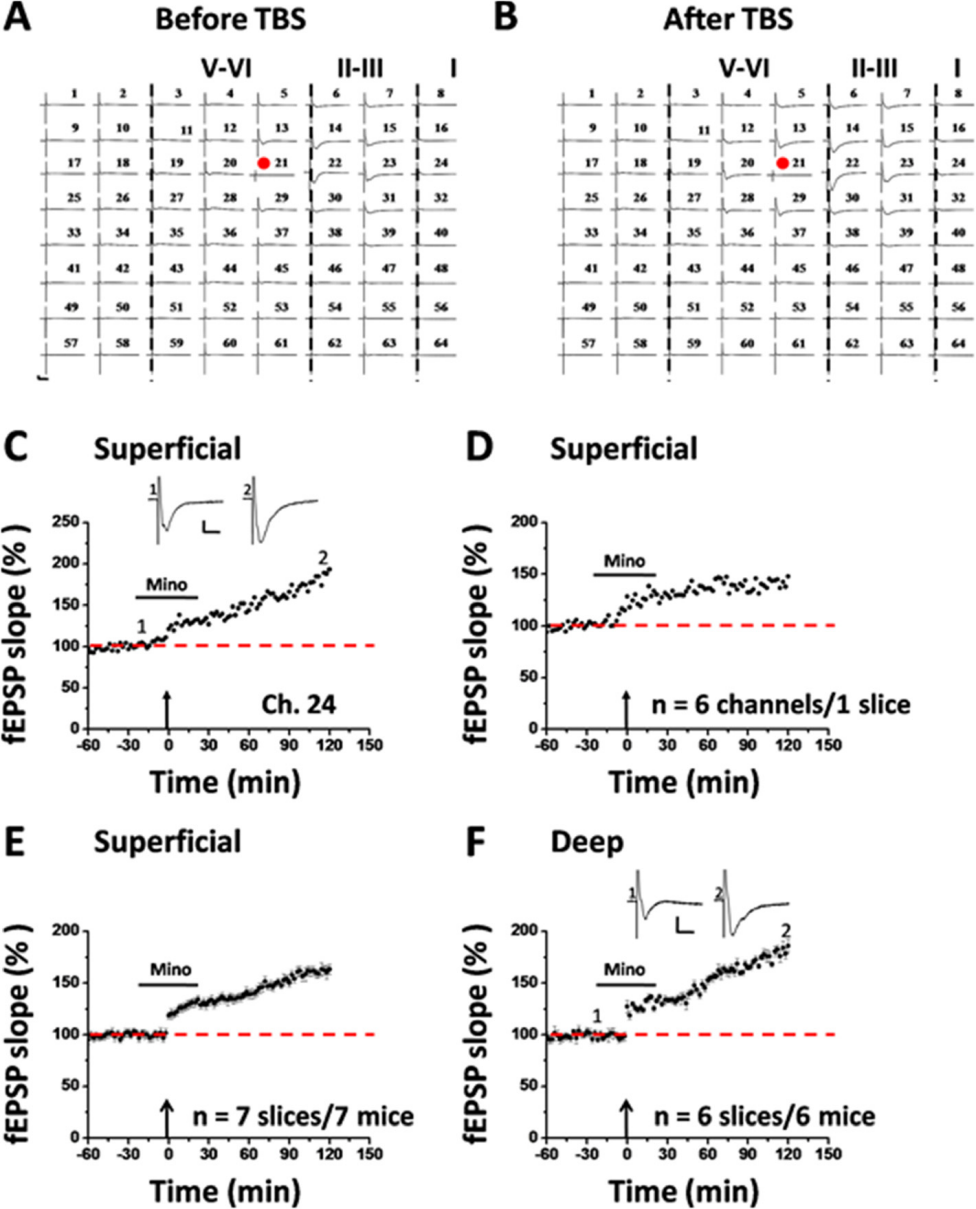
Minocycline has no effect on post-LTP induction in the ACC. **A** and **B**: one sample of 64-channel recordings of cingulate post-LTP induced in the presence of minocycline (Mino, 100 μM). A, baseline; B, 2 h after TBS. After stabilizing the baseline responses for 30 min, minocycline was bath applied from 30 min before till 30 min after delivery of TBS to the deep layer. Minocycline could not prevent the post-LTP induction. The red filled circle denotes the stimulated channel (Ch. 21). Vertical lines demarcate specific layers. **C**: results of one channel (Ch. 24) showing the normal induction of post-LTP by TBS. **D**: summary of averaged data from 6 superficial channels of 1 slice. **E**: pooled data of the superficial layer from 7 slices of 7 mice. **F**: pooled data of the deep layer from 6 slices of 6 mice. Insets in C and F are sample traces at the time points indicated by numbers in the graph. Calibration: 100 μV, 10 ms. Arrows in C-F indicate starting point of TBS delivery. Error bars in E and F represent SEM.

To determine whether minocycline can block post-LTP in the ACC, we bath applied minocycline (100 μM) 30 min prior to the TBS delivery. The drug was washed out 30 min after TBS and LTP was monitored for another 2 h. Here, it is noteworthy that minocycline used in this dose has been demonstrated to effectively block microglia activation in previous studies [22,37]. One representative 64-channel recording is illustrated in Figure 3A (before TBS) and Figure 3B (2 h after TBS) for the minocycline-treated group. We found that TBS still induced a long-lasting potentiation of fEPSP within the ACC network. Analysis of one single channel (Ch. 24) in the superficial layer revealed that the response was potentiated to 193.1% of baseline at 2 h after TBS (Figure 3C). The averaged data from 6 superficial channels for this slice is presented in Figure 3D (147.6% of baseline). Pooled data from 7 slices of 7 mice are shown in Figure 3E (163.2 ± 4.6% of baseline, *P* < 0.001, paired t-test). Similar results were obtained when analyzing the channels located in the deep layer of the ACC. As illustrated in Figure 3F, pre-treatment with minocycline failed to block the post-LTP induction (185.7 ± 6.1% of baseline, n = 6 slices/6 mice, *P* < 0.001, paired t-test).

We also performed network analysis of the post-LTP distribution maps in minocycline-exposed ACC slices. Bath application of minocycline affected neither the activation map nor the post-LTP map in the ACC (Figure 2C and D). We found that a total of 104 channels (mean ± SEM: 14.9 ± 0.5) were activated from 7 slices, and 73 channels (mean ± SEM: 10.4 ± 0.6) underwent post-LTP. We did not find any significant difference in the induction ratio of post-LTP between control and minocycline-treated group (control vs. minocycline: 80.8 ± 3.3% vs. 70.4 ± 4.0%).

### Multi-channel recordings of pre-LTP in the ACC circuit

**Figure 4:**
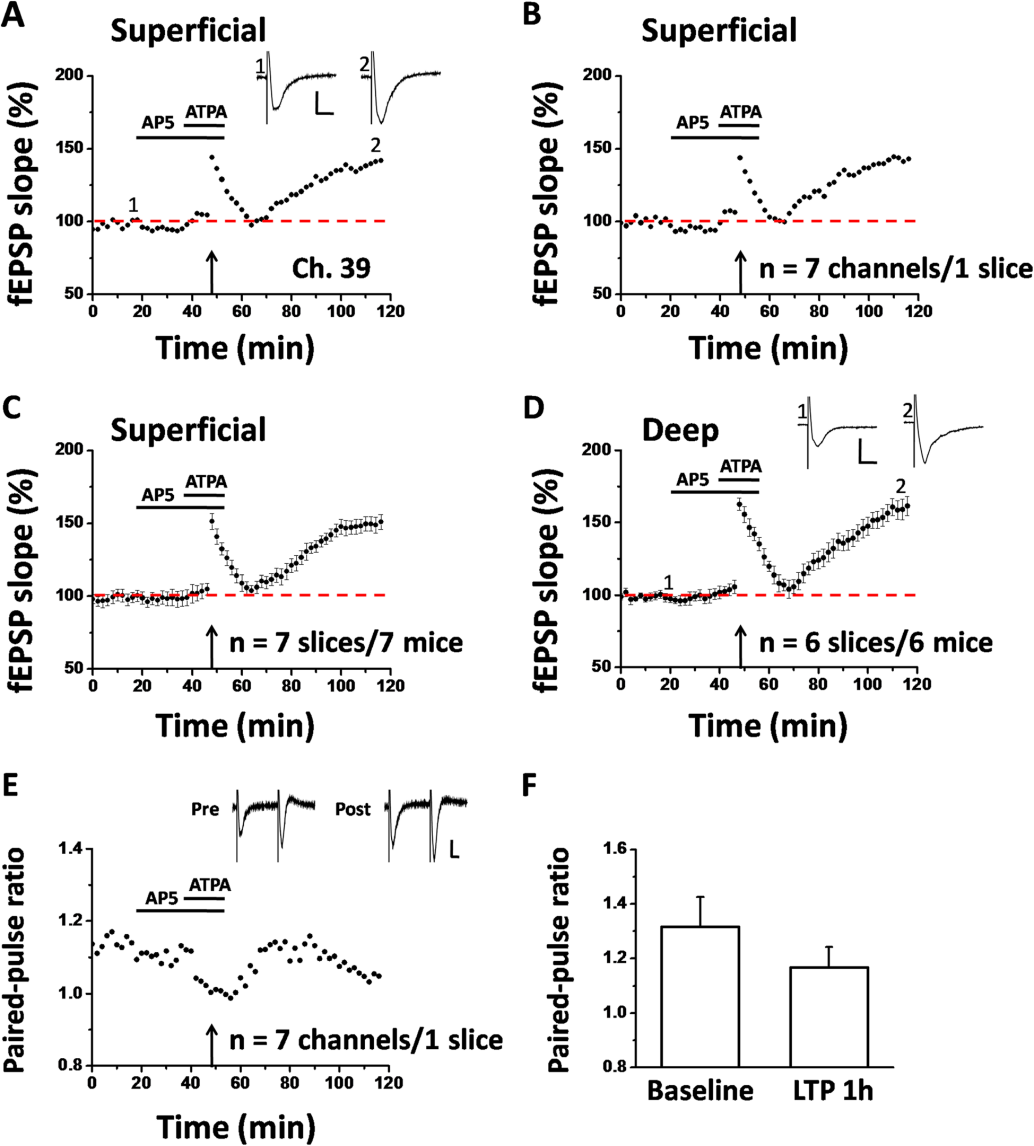
Multi-channel recordings of pre-LTP in the adult mouse ACC. **A**: results of one pre-LTP-showing channel (Ch. 39) from the superficial layer of one ACC slice. After the baseline response was stabilized for 20 min, a LFS protocol (2 Hz for 2 min) was applied in the presence of the NMDA receptor antagonist (AP-5, 50 μM, 38 min) and a GluK1-containing kainate receptor agonist (ATPA, 1 μM, 18 min). The synaptic potentiation could last for at least 1 h. **B**: summary of averaged data from 7 superficial channels of the same slice. **C**: pooled data for the superficial layer from 7 slices of 7 mice. **D**: summarized data for the deep layer of the ACC from 6 slices of 6 mice. **E**: PPR was decreased 1 h after the LFS + ATPA in one slice (averaged data of 7 channels). **F**: bar histogram showing the pooled data of PPR from 7 slices of 7 mice. Insets in A and D show representative fEPSP traces at time points indicated by the numbers in the graph. Sample traces in E are paired-pulse responses before (Pre) and 1 h after pre-LTP induction (Post). Calibration: 50 μV, 10 ms. Horizontal bars in A-E denote the period of AP5 or ATPA application as indicated. Arrows in A-E indicate the LFS application. Error bars in C, D and F represent SEM.

To explore the possibility of pre-LTP induction in the adult mouse ACC, we designed the following protocol according to the previous studies [27]. After the baseline synaptic responses were stabilized for 20 min, AP5 (50 μM) was bath applied to prevent the activation of NMDA receptors. Twenty minutes later, a GluK1-containing kainate receptor agonist (ATPA, 1 μM) was infused for 18 min, but still in the presence of AP5. A LFS protocol (2 Hz, 2 min) was given at 8 min after the onset of ATPA application, after which ATPA was washed out 8 min later [27]. Figure 4A and B illustrate the single-channel data (Ch. 39, 141.6% of baseline at 1 h after the LFS + ATPA protocol) within one example slice and averaged results of 7 channels in the superficial layer of the same slice (143.0% of baseline), respectively. Pooled results from a series of similar experiments are summarized in Figure 4C, with the mean fEPSP slope increasing to 150.9 ± 5.1% of baseline at the end of the recording (n = 7 slices/7 mice, *P* < 0.001, paired t-test). Synaptic responses in the deep layer of the ACC were also potentiated (161.5 ± 6.6% of baseline, n = 6 slices/6 mice, *P* < 0.001, paired t-test, Figure 4D).

We next wanted to confirm changes in synaptic responses to paired-pulse stimulation. We calculated the changes in the ratio of paired-pulse responses (or called PPR) and found that ATPA combined with LFS significantly reduced the PPR at 50 ms interval (baseline vs. 1 h after LFS + ATPA protocol: 1.13 vs. 1.05, n = 7 channels/1 slice, Figure 4E). Pooled data from 7 slices are shown in Figure 4F, showing the similar reduction of PPR by the LTP induction (baseline vs. 1 h after pre-LTP protocol: 1.32 ± 0.11 vs. 1.17 ± 0.07). These data indicate that LTP evoked by LFS + ATPA combination represents a distinct form of LTP that is expressed presynaptically.

**Figure 5:**
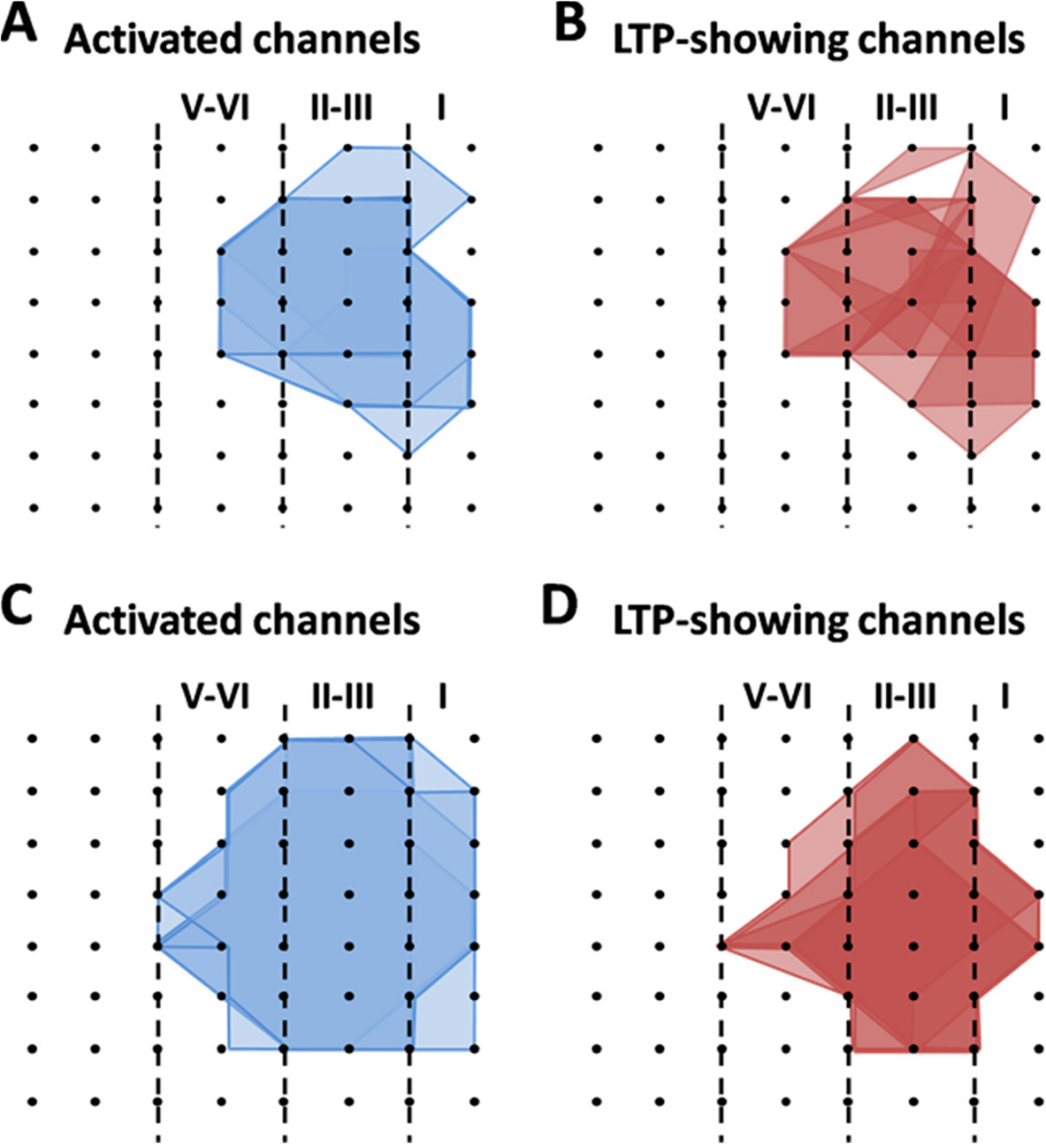
Spatial analysis of pre-LTP distribution in the ACC. **A** and **B**: polygonal diagram of the channels that are activated in the baseline (blue, A) and that undergo pre-LTP (red, B) in 7 slices from 7 mice. Vertical lines denote the layers in the ACC slice. **C** and **D**: spatial analysis of the effects of minocycline on pre-LTP distribution maps in the ACC. Shown are the polygonal graphs of activated (blue, C) and pre-LTP-occurring (red, D) channels among the cingulate network when LFS + ATPA is applied in the presence of minocycline (n = 5 slices/5 mice). Spatial distribution of pre-LTP-showing channels is not significantly altered by minocycline.

Finally, we examined the spatial distribution of pre-LTP-showing channels across the ACC circuit. The number of activated channels and pre-LTP-occurring channels were counted for each slice and the grouped data is plotted in Figure 5A and B. Among the 7 slices analyzed, 95 channels (mean ± SEM: 13.6 ± 1.7) were activated and thus showing typical fEPSP in the baseline condition, while 70 channels (mean ± SEM: 10.0 ± 1.9) exhibited pre-LTP, with an induction ratio being 71.8 ± 8.3%. Similar to the post-LTP distribution map, pre-LTP-showing channels were also found in both layer II-III and V-VI of the ACC slice (Figure 5B).

### Minocycline does not affect pre- LTP induction in the ACC

**Figure 6:**
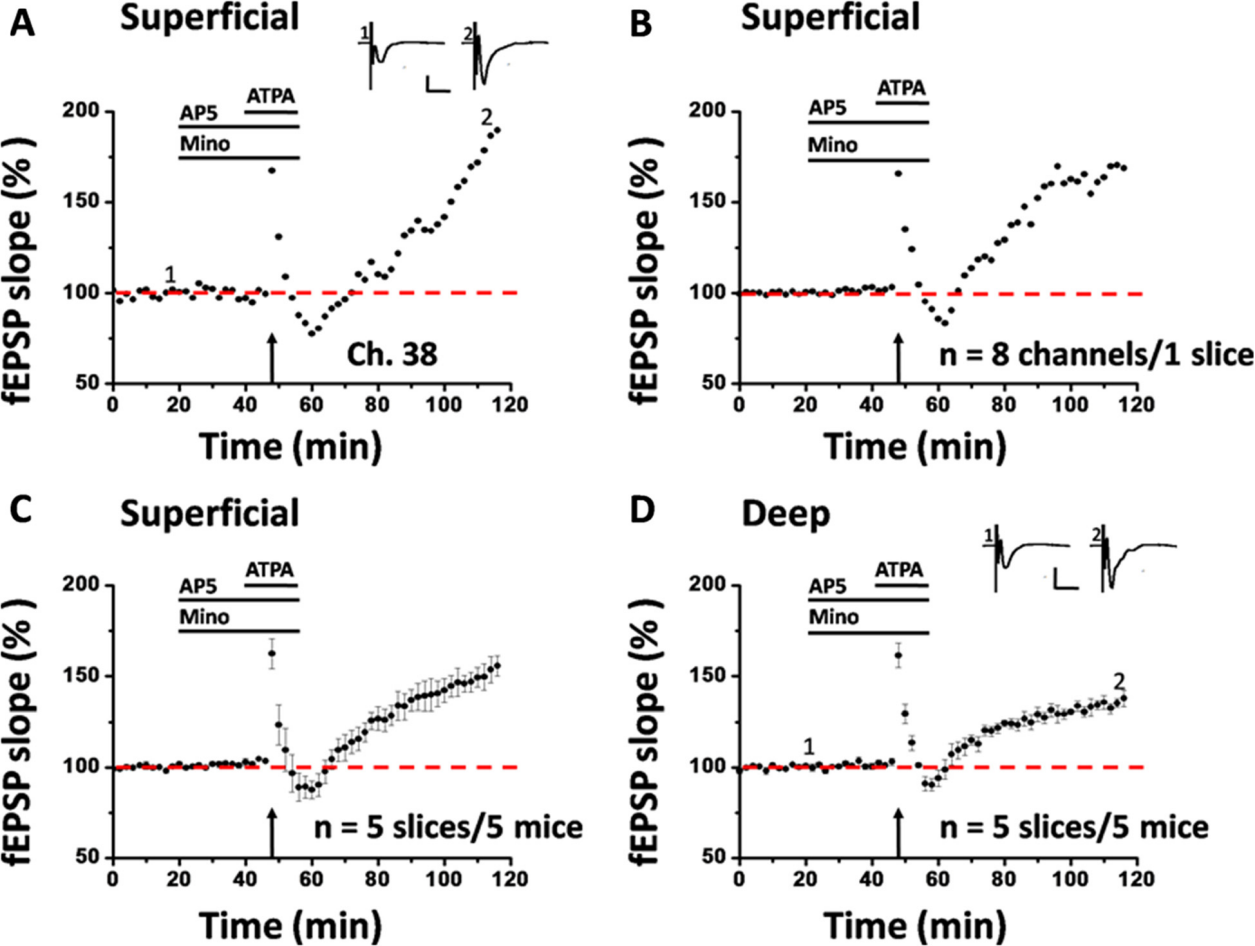
Minocycline does not affect pre-LTP induction in the ACC. **A**: results of one channel (Ch. 38) from the superficial layer of one ACC slice. After the baseline response was stabilized for 20 min, an identical LFS + ATPA protocol was applied as that in Fig. 4 but now in the presence of minocycline (Mino, 100 μM). However, minocycline could not block the pre-LTP induction. **B**: summary of averaged data from 8 superficial channels of the same slice. **C**: pooled data for the superficial layer from 5 slices of 5 mice. **D**: summarized data for the deep layer from 5 slices of 5 mice. Insets in A and D show representative fEPSP traces at time points indicated by the numbers in the graph. Calibration: 100 μV, 10 ms. Horizontal bars in A-D denote the period of AP5 or ATPA or minocycline application as indicated. Arrows in A-D indicate the LFS application. Error bars in C and D represent SEM.

Since pre-LTP and post-LTP represent two different forms of LTP mediating different functions in the ACC [29], we next asked whether minocycline may block pre-LTP induction in the ACC of normal adult mice. We applied minocycline (100 μM, 38 min) together with AP5 for the entire duration of the pre-LTP induction protocol. Interestingly, we found that minocycline had no effect on pre-LTP either. Delivery of LFS + ATPA to the deep layer of the ACC slice still resulted in a prolonged synaptic potentiation in the presence of minocycline (single channel: Ch. 38, 214.1% of baseline, Figure 6A; averaged 8 channels from 1 slice: 167.8% of baseline, Figure 6B; pooled data: 160.6 ± 8.0% of baseline, n = 5 slices/5 mice, *P* < 0.001, paired t-test, Figure 6C). We also analyzed the changes in the deep layer and found that the fEPSP slope was equally potentiated to 141.2 ± 6.4% of baseline, n = 5 slices/ 5 mice, Figure 6D). The spatial map of pre-LTP was also unaltered by minocycline (n = 5 slices/ 5 mice, Figure 5C and D). There are 93 LTP-showing channels (mean ± SEM: 12.6 ± 2.0) out of 136 fEPSP-showing channels (mean ± SEM: 17.2 ± 1.2). No significant difference was detected in the induction ratio of pre-LTP between control and minocycline-exposed group (control vs. minocycline: 71.8 ± 8.3% vs. 73.3 ± 5.6%).

## Discussion

It has been well established that synaptic plasticity such as LTP and LTD plays important roles in various physiological functions and pathological conditions including learning, memory, chronic pain and addiction [17,19,38-40]. Recent studies have suggested that glial cells may contribute to this plasticity, especially under pathological conditions [23,41,42] (see Additional file 2: Table S2). However, most of these studies are indirect or suggestive. In the present study, we used a 64-channel multi-electrode array recording system to examine the potential role of microglia in LTP induction in the ACC. We found that bath application of minocycline, a potent microglia inhibitor [11,12], failed to affect either post-LTP or pre-LTP in the ACC of normal adult mice. The spatial properties of post-LTP or pre-LTP distribution were also unaltered by minocycline, suggesting a rather minor role of microglial cells in cingulate synaptic plasticity. These results, together with our previous publications [15,26], reinforce the importance of neuronal changes in cortical plasticity [19,40].

Several lines of evidence have been accumulated to demonstrate the pivotal role of microglia in spinal pain processing [2,43,44]. Under either inflammatory or neuropathic pain conditions, glial cells undergo a conversion from resting surveillance state to active state in the spinal cord [6,45]. When activated, microglia can release a number of proinflammatory mediators or cytokines, which in turn contribute to the pathophysiology of nociceptive sensitization [8,10,46]. Compared to the spinal cord, however, relatively little is known about the contribution of glial cells in supraspinal level of pain processing (Additional file 1: Table S1). Among the few studies investigating this issue, controversy still exists, with some papers showing the glial involvement in pain-evoked sensory hypersensitivity [14] and emotional aversion [13,47], and other papers showing no critical role of microglia in cortical pain perception [15].

It is generally believed that sensory plasticity taking place in the ascending sensory pathways is a well-defined cellular model and endpoint measurement of chronic pain [18,48]. For example, persistent nociceptive stimuli can result in a LTP-like enhancement of synaptic transmission in the ACC [49,50], and preventing or erasing cingulate LTP produces analgesic effects in animal pain models [35,51]. In the present study, we found that minocycline had no effect on either the induction probability or spatial distribution of TBS-evoked post-LTP in the ACC under normal condition, indicating no crucial involvement of microglia in cortical plasticity that is believed to be important for chronic pain. Consistent with these observations, our previous work revealed no significant change in the density and activation state of microglia in the ACC under neuropathic pain conditions [15]. These negative results are in contrast with previous studies in the spinal cord dorsal horn, where microglia is shown to be necessary for spinal LTP induction both *in vivo* and *in vitro* [20-22,42]. It is possible that the roles of microglia in chronic pain are highly limited at the level of the spinal cord. In addition, these results highlight the necessity of using cortical plasticity as an additional index for analgesic drug evaluation and screening [18,48].

Compared to post-LTP, much less information is now available on pre-LTP in the neocortex. Recently, we report the first demonstration of pre-LTP in the ACC using both whole-cell patch-clamp and 64-channel multisite recordings [27,29]. We found that combinations of LFS and a GluK1 receptor agonist (ATPA) could induce a novel form of metabotropic glutamate receptor-dependent LTP that is expressed presynaptically in both superficial and deep layers of the ACC [27]. In this study, we also investigated the role of microglia in this form of cingulate pre-LTP and obtained similar results with post-LTP. The lack of any effect of minocycline is not likely due to the insufficient dose applied during the experiment, because previous studies using the same dose of the drug got the positive results *in vitro* [22,52]. Importantly, our recent work show that pre-LTP in the ACC is more likely related to pain-evoked anxiety than the sensory aspect of pain perception [29]. Thus, our results indicate that microglia equally has a minor role in emotional comorbidities of chronic pain. It will be interesting in future studies to further test this hypothesis in more detail.

In summary, the present study provides the initial investigation of the effect of minocycline on cortical plasticity in the ACC of normal adult mice. Neither post-LTP nor pre-LTP is affected by the drug in both temporal and spatial domains. Our findings suggest that minocycline does not affect cingulate plasticity and neurons are the major players in pain-related cortical plasticity.

## Additional files

Additional file 1: Table S1. Summary of physiological and pathological functions contributed by microglia in the brain. Additional file 2: Table S2. Summary of previous studies on microglia and synaptic plasticity in the brain.
