# Supplementary material for: Minocycline does not affect long-term potentiation in the anterior cingulate cortex of normal adult mice
Source: Mol Pain. 2015 May 2;11:25. doi: 10.1186/s12990-015-0025-2 (PMC4464617; doi:10.1186/s12990-015-0025-2)
Supplement: Additional file 1: Table S1. — Summary of physiological and pathological functions contributed by microglia in the brain. [file 12990_2015_25_MOESM1_ESM.docx]

**Table S1 Summary of physiological and pathological functions contributed by microglia in the brain**

| **Brain function** | **Manipulation** | | | **Brain regions** | **Effects Reference** | | |  |  |
| --- | --- | --- | --- | --- | --- | --- | --- | --- | --- |
| **Pain** | |  |  | | |  |  | | |
| Spare nerve injury | | Pharmacology | Prefrontal cortex | | | AMPA receptor upregulation and caspase 3 release in microglia contribute to neuropathic mechanical allodynia | ([Giordano et al. 2012](#_ENREF_7)) | | |
| Spinal nerve ligation | | Minocycline | Prefrontal cortex | | | Minocycline attenuates neuropathic pain behavior in a manner dependent on the presence of a depression-like phenotype | ([Burke et al. 2014](#_ENREF_3)) | | |
| Formalin-induced  conditioned place aversion | | p38 MAPK inhibitor | ACC | | | p38 MAPK activation in the microglia contributes to pain-related negative emotion | ([Cao et al. 2014](#_ENREF_4)) | | |
| Spinal cord injury | | p38 MAPK inhibitor | ACC | | | Oral treatment of a p38 MAPK inhibitor reduces pain-evoked aversion accompanied by a reduction of microglia in the ACC | ([Galan-Arriero et al. 2014](#_ENREF_6)) | | |
| Visceral pain | | TLR4 deficient mice  TLR4 antagonist | Prefrontal cortex | | | Microglia modulates chronic stress-induced visceral hypersensitivity through TLR4 | ([Tramullas et al. 2014](#_ENREF_13)) | | |
| **Learning and memory** | |  |  | | |  |  | |  |
| Radial arm water maze | | Tg-RAGE mice  Tg-mAPP mice | Hippocampus and cortex | | | RAGE signaling in microglia accelerates the deterioration of spatial learning and memory induced by accumulation of Aβ | ([Fang et al. 2010](#_ENREF_5)) | | |
| Fear conditioning | | Minocycline | Hippocampus | | | Microglia is involved in the memory impairment in neonatally infected rats | ([Williamson et al. 2011](#_ENREF_14)) | | |
| Novel object recognition | | Minocycline | Whole brain | | | Activated microglia mediates short-term memory deficits in a mouse model of OA | ([Azevedo et al. 2013](#_ENREF_2)) | | |
| Water maze | | Minocycline | Hippocampus | | | Minocycline treatment improves the water maze learning | ([Kohman et al. 2013](#_ENREF_8)) | | |
| Fear conditioning  Motor learning  Novel object recognition | | CX3CR1^CreER^  mice | Hippocampus and cortex | | | Microglia promotes learning-related synapse formation through BDNF signaling | ([Parkhurst et al. 2013](#_ENREF_9)) | | |
| Water maze  Fear conditioning  Olfactory memory | | CX3CR1 deficient  mice or CX3CR1 blocking antibodies | Hippocampus and olfactory bulb | | | Microglia is only related to the regulation of memory by environmental enrichment in the hippocampus | ([Reshef et al. 2014](#_ENREF_10)) | | |
| **Addiction** | |  |  | | |  |  |  |  |
| Ethanol | | Minocycline | Whole brain | | | Minocycline reduces ethanol intake in a free choice voluntary drinking mouse model | ([Agrawal et al. 2011](#_ENREF_1)) | | |
| Morphine | | Ibudilast | Nucleus accumbens | | | Neonatal handling decreases drug-induced instatement in adulthood through blocking microglia activation | ([Schwarz et al. 2011](#_ENREF_12)) | | |
| Morphine | | Minocycline  p38 MAPK inhibitor | Nucleus accumbens | | | p38 signaling in the microglia plays an important role in the acquisition and maintenance but not the expression of morphine-induced CPP | ([Zhang et al. 2012](#_ENREF_15)) | | |
| Morphine | | Minocycline  Ibudilast | Nucleus accumbens | | | Adolescent morphine pre-exposure affects long-term microglial function, altering the risk of drug-induced instatement in adulthood | ([Schwarz and Bilbo 2013](#_ENREF_11)) | | |

Notes: ACC, anterior cingulate cortex; BDNF, brain-derived neurotropic receptor; CPP, conditioned place preference; CX3CR1, CX3CL1 receptor; MAPK, mitogen-activated protein kinase; mAPP, mutant amyloid precursor protein; OA, oculoleptomeningeal amyloidosis; RAGE; receptor for advanced glycation end products; Tg, transgenic; TLR4, toll-like receptor 4.

Agrawal RG, Hewetson A, George CM, Syapin PJ, and Bergeson SE. Minocycline reduces ethanol drinking. Brain Behav Immun*.* 2011;25 Suppl 1: S165–169.

Azevedo EP, Ledo JH, Barbosa G, Sobrinho M, Diniz L, Fonseca AC, Gomes F**,** et al. Activated microglia mediate synapse loss and short-term memory deficits in a mouse model of transthyretin-related oculoleptomeningeal amyloidosis. Cell Death Dis*.* 2013;4: e789.

Burke NN, Kerr DM, Moriarty O, Finn DP, and Roche M. Minocycline modulates neuropathic pain behaviour and cortical M1-M2 microglial gene expression in a rat model of depression. Brain Behav Immun. 2014;42: 147–56.

Cao H, Zang KK, Han M, Zhao ZQ, Wu GC, and Zhang YQ. Inhibition of p38 mitogen-activated protein kinase activation in the rostral anterior cingulate cortex attenuates pain-related negative emotion in rats. Brain Res Bull*.* 2014;107: 79–88.

Fang F, Lue LF, Yan S, Xu H, Luddy JS, Chen D, et al. RAGE-dependent signaling in microglia contributes to neuroinflammation, Abeta accumulation, and impaired learning/memory in a mouse model of Alzheimer's disease. FASEB J. 2010;24: 1043–55.

Galan-Arriero I, Avila-Martin G, Ferrer-Donato A, Gomez-Soriano J, Bravo-Esteban E, and Taylor J. Oral administration of the p38alpha MAPK inhibitor, UR13870, inhibits affective pain behavior after spinal cord injury. Pain. 2014;155: 2188–98.

Giordano C, Cristino L, Luongo L, Siniscalco D, Petrosino S, Piscitelli F, et al. TRPV1-dependent and -independent alterations in the limbic cortex of neuropathic mice: impact on glial caspases and pain perception. Cerebr Cortex*.* 2012;22: 2495–518.

Kohman RA, Bhattacharya TK, Kilby C, Bucko P, and Rhodes JS. Effects of minocycline on spatial learning, hippocampal neurogenesis and microglia in aged and adult mice. Behav Brain Res*.* 2013;242: 17–24.

Parkhurst CN, Yang G, Ninan I, Savas JN, Yates JR, 3rd, Lafaille JJ, et al. Microglia promote learning-dependent synapse formation through brain-derived neurotrophic factor. Cell. 2013*;*155: 1596–609.

Reshef R, Kreisel T, Beroukhim Kay D, and Yirmiya R. Microglia and their CX3CR1 signaling are involved in hippocampal- but not olfactory bulb-related memory and neurogenesis. Brain Behav Immun*.* 2014;41: 239–50.

Schwarz JM, and Bilbo SD. Adolescent morphine exposure affects long-term microglial function and later-life relapse liability in a model of addiction. J Neurosci. 2013;33: 961–71.

Schwarz JM, Hutchinson MR, and Bilbo SD. Early-life experience decreases drug-induced reinstatement of morphine CPP in adulthood via microglial-specific epigenetic programming of anti-inflammatory IL-10 expression. J Neurosci. 2011;31: 17835–47.

Tramullas M, Finger BC, Moloney RD, Golubeva AV, Moloney G, Dinan TG, and Cryan JF. Toll-like receptor 4 regulates chronic stress-induced visceral pain in mice. Biol Psychiatr. 2014;76: 340–8.

Williamson LL, Sholar PW, Mistry RS, Smith SH, and Bilbo SD. Microglia and memory: modulation by early-life infection. J Neurosci*.* 2011;31: 15511–21.

Zhang XQ, Cui Y, Cui Y, Chen Y, Na XD, Chen FY,et al. Activation of p38 signaling in the microglia in the nucleus accumbens contributes to the acquisition and maintenance of morphine-induced conditioned place preference. Brain Behav Immun*.* 2012;26: 318–25.
