# Supplementary material for: Minocycline does not affect long-term potentiation in the anterior cingulate cortex of normal adult mice
Source: Mol Pain. 2015 May 2;11:25. doi: 10.1186/s12990-015-0025-2 (PMC4464617; doi:10.1186/s12990-015-0025-2)
Supplement: Additional file 2: Table S2. — Summary of previous studies on microglia and synaptic plasticity in the brain. [file 12990_2015_25_MOESM2_ESM.docx]

**Table 2. Summary of previous studies on microglia and synaptic plasticity in the brain**

| **CNS region** | **Species** | **Method** | **Plasticity type** | **Key findings** | | | | | | **Reference** |
| --- | --- | --- | --- | --- | --- | --- | --- | --- | --- | --- |
| Hippocampus CA1 region | Mouse | fEPSP | LTP | Enhanced microglia activation contributes to the LTP deficits in membrane glycoprotein CD200 deficient mice | | | | | ([Costello et al. 2011](#_ENREF_3)) | |
| Hippocampus CA1 region | Mouse | fEPSP | LTP | IL-1β expression in the microglia leads to enhanced LTP in EAE mice | | | | | ([Nistico et al. 2013](#_ENREF_8)) | |
|  |  |  |  |  | | | | |  | |
| Hippocampus CA1 region | Rat | EPSC | LTP | Activation of CX3CR1 in the microglia contributes to Aβ-induced inhibition of LTP | | | | | ([Wu et al. 2013](#_ENREF_10)) | |
| Hippocampus CA1 region | Mouse | In vivo EPSP | LTP | Microglia inhibits LTP induction through IKKβ | | | | | ([Kyrargyri et al. 2014](#_ENREF_7)) | |
| Hippocampus CA1 region | Rat and mouse | fEPSP | LTD | LPS and hypoxia induce LTD that requires microglial CR3 activation | | | | | ([Zhang et al. 2014](#_ENREF_11)) | |
| Hippocampus CA1 region and dentate gyrus | Rat and mouse | fEPSP | LTP | Microglia is involved in Aβ-mediated inhibition of NMDA receptor-dependent LTP in the dentate gyrus | | | | | ([Wang et al. 2004](#_ENREF_9)) | |
| Dentate gyrus | Rat | In vivo EPSP | LTP | Microglia is involved in age-related attenuation of LTP | | | | | | ([Griffin et al. 2006](#_ENREF_5)) |
| Spinal cord dorsal horn | Rat | In vivo EPSP | LTP | ATP may activate p38 MAPK in microglia to induce spinal LTP | | | | | | ([Gong et al. 2009](#_ENREF_4)) |
| Spinal cord dorsal horn | Rat | In vivo EPSP | LTP and LTD | SFK in the microglia determines the direction of synaptic plasticity | | | | | | ([Zhong et al. 2010](#_ENREF_12)) |
| Spinal cord dorsal horn | Rat | In vivo EPSP and fEPSP | LTP | The P2X7 receptors in microglia play a pivotal role in the induction of spinal LTP | | | | | | ([Chu et al. 2010](#_ENREF_1)) |
| Spinal cord dorsal horn | Rat | In vivo EPSP | LTP | | Microglia is involved in BDNF-induced spinal LTP | | ([Zhou et al. 2011](#_ENREF_13)) | | | |
| Spinal cord dorsal horn | Rat | In vivo EPSP | LTP | | | The IL-18 signaling pathway in microglia is involved in the induction of spinal LTP | | ([Chu et al. 2012](#_ENREF_2)) | | |
| Spinal cord dorsal horn | Rat | In vivo EPSP and fEPSP | LTP | | | Spinal microglia is necessary for LTP induction in vitro and in vivo | | ([Gruber-Schoffnegger et al. 2013](#_ENREF_6)) | | |
| Entorhinal cortex | Mouse | fEPSP | LTD | | | Microglia RAGE signaling contributes to Aβ-induced LTD impairment in entorhinal cortex | | (Origlia et al., 2010) | | |
| ACC | Mouse | Multi-channel fEPSP | LTP | | | Minocycline has no effect on either post-LTP or pre-LTP induction in the ACC | | The present study | | |

Notes: ACC, anterior cingulate cortex; ATP, adenosine triphosphate; BDNF, brain-derived neurotrophic factor; CR3, complement receptor 3; CX3CR1, CX3CL1 receptor; EAE, experimental autoimmune encephalomyelitis; EPSC, excitatory postsynaptic current; fEPSP, field excitatory postsynaptic potential; IKKβ, the inhibitor of κ B kinase β; IL-1β,interleukin-1β; IL-18, interleukin-18; LPS, lipopolysaccharide; LTD, long-term depression; LTP, long-term potentiation; MAPK, mitogen-activated protein kinase; RAGE, receptor for advanced glycation end products; SFK, src-family kinase.

Chu YX, Zhang Y, Zhang YQ, Zhao ZQ. Involvement of microglial P2X7 receptors and downstream signaling pathways in long-term potentiation of spinal nociceptive responses. Brain Behav Immun. 2010;24: 1176–89.

Chu YX, Zhang YQ, Zhao ZQ. Involvement of microglia and interleukin-18 in the induction of long-term potentiation of spinal nociceptive responses induced by tetanic sciatic stimulation. Neurosci Bull. 2012;28: 49–60.

Costello DA, Lyons A, Denieffe S, Browne TC, Cox FF, and Lynch MA. Long term potentiation is impaired in membrane glycoprotein CD200-deficient mice: a role for Toll-like receptor activation. J Biol Chem. 2011;286: 34722–32.

Gong QJ, Li YY, Xin WJ, Zang Y, Ren WJ, Wei XH, et al. ATP induces long-term potentiation of C-fiber-evoked field potentials in spinal dorsal horn: the roles of P2X4 receptors and p38 MAPK in microglia. Glia. 2009;57: 583–91.

Griffin R, Nally R, Nolan Y, McCartney Y, Linden J, Lynch MA. The age-related attenuation in long-term potentiation is associated with microglial activation. J Neurochem. 2006;99: 1263–72.

Gruber-Schoffnegger D, Drdla-Schutting R, Honigsperger C, Wunderbaldinger G, Gassner M, and Sandkuhler J. Induction of thermal hyperalgesia and synaptic long-term potentiation in the spinal cord lamina I by TNF-alpha and IL-1beta is mediated by glial cells. J Neurosci. 2013;33: 6540–51.

Kyrargyri V, Vega-Flores G, Gruart A, Delgado-Garcia JM, and Probert L. Differential contributions of microglial and neuronal IKKbeta to synaptic plasticity and associative learning in alert behaving mice. Glia*.* 2015;63: 549–66.

Nistico R, Mango D, Mandolesi G, Piccinin S, Berretta N, Pignatelli M, et al. Inflammation subverts hippocampal synaptic plasticity in experimental multiple sclerosis. PloS One*.* 2013;8: e54666.

Origlia N1, Bonadonna C, Rosellini A, Leznik E, Arancio O, Yan SS, et al**.** Microglial receptor for advanced glycation end product-dependent signal pathway drives beta-amyloid-induced synaptic depression and long-term depression impairment in entorhinal cortex. J Neurosci. 2010;30:11414–25.

Wang Q, Rowan MJ, and Anwyl R. Beta-amyloid-mediated inhibition of NMDA receptor-dependent long-term potentiation induction involves activation of microglia and stimulation of inducible nitric oxide synthase and superoxide. J Neurosci. 2004;24: 6049–56.

Wu J, Bie B, Yang H, Xu JJ, Brown DL, and Naguib M. Suppression of central chemokine fractalkine receptor signaling alleviates amyloid-induced memory deficiency. Neurobiol Aging. 2013;34: 2843–52.

Zhang J, Malik A, Choi HB, Ko RW, Dissing-Olesen L, and MacVicar BA. Microglial CR3 activation triggers long-term synaptic depression in the hippocampus via NADPH oxidase. Neuron. 2014;82: 195–207.

Zhong Y, Zhou LJ, Ren WJ, Xin WJ, Li YY, Zhang T, et al. The direction of synaptic plasticity mediated by C-fibers in spinal dorsal horn is decided by Src-family kinases in microglia: the role of tumor necrosis factor-alpha. Brain Behav Immun. 2010;24: 874–80.

Zhou LJ, Yang T, Wei X, Liu Y, Xin WJ, Chen Y, et al. Brain-derived neurotrophic factor contributes to spinal long-term potentiation and mechanical hypersensitivity by activation of spinal microglia in rat. Brain Behav Immun. 2011;25: 322–34.
